# Approche diagnostique d'une dégénérescence maculaire occulte par OCT de type “spectral-domain”: cas Clinique

**DOI:** 10.11604/pamj.2015.22.61.4850

**Published:** 2015-09-22

**Authors:** Aziz El Ouafi, Med El Mellaoui, Abdelkader Laktaoui

**Affiliations:** 1 Service Ophtalmologie Hopital Militaire Mohammed IV, Meknès, Maroc

**Keywords:** Dégénérescence maculaire occulte, OCT, images, Occult macular degeneration, OCT, images

## Abstract

Le diagnostic de la dégénérescence maculaire occulte est difficile. Il pourrait être facilité grâce aux nouvelles techniques d'acquisition des images par S-D OCT. L'objectif de ce travail est de discuter de l'intérêt de l'OCT à haute résolution dans le diagnostic d'une dégénérescence maculaire occulte.

## Introduction

La dégénérescence maculaire occulte est une pathologie dont l’étiologie est encore méconnue bien qu'il semble qu'il y ait une transmission héréditaire de la maladie. Le diagnostic repose sur la mise en évidence d'anomalies à l'ERG multifocal alors que l'ERG plein champ, la fluoroangiographie et l'examen du FO sont tout à fait normaux. L'OCT spectralis apporte des informations utiles au diagnostic.

## Patient et observation

**Figure 1:**
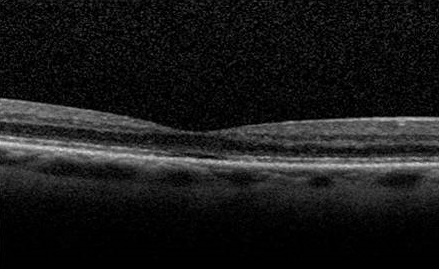
Angio et OCT de l'oeil droit

**Figure 2:**
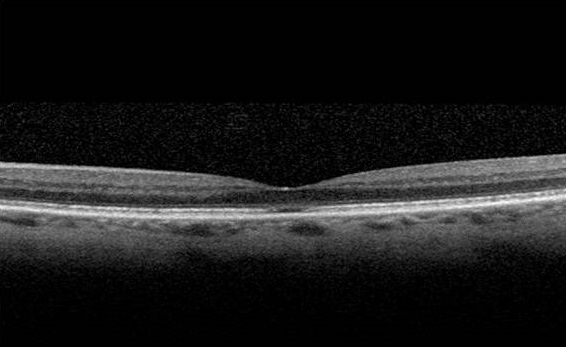
Angio et OCT de l'oeil gauche montrant la néovascularisation choroidienne

Une femme âgée de 65 ans est suivie depuis 06 ans pour une dégénérescence maculaire occulte (DMO). Elle avait été référée pour une baisse d'acuité visuelle ainsi qu'une dyschromatopsie bilatérale progressive sans explication étiologique. Tout au long de son suivi, aucun changement dans le fond d'oeil (FO) n'a été identifié. L'acuité visuelle de loin était mesurée en 2006 à 5/10 à l'OD et 09/10 à l'OG et en 2013 à 2/10 à l'OD et 5/10 à l'OG. Il n'y a pas de déficit afférent pupillaire. La vision des couleurs est altérée bilatéralement à l'Ischihara. L'examen du fond d'oeil et la fluoro angiographie sont normaux. Des scotomes centraux bilatéraux sont présents. Des clichés auto fluorescents du fond d'oeil ont été réalisés et montrent un background fluorescent homogène qui s'avère normal. Pour finir, l’épaisseur fovéolaire à l'OCT ( tomgraphie par cohérence optique) en “time-domain” (T-D OCT) est estimée à 174 um à l'OD et 160 um à l'OG ce qui reste dans les limites de la normale (Figure 1). Par contre, l'OCT de type “spectral-domain” (S-D OCT) met clairement en évidence un défect rétrofovéolaire au niveau de la jonction entre les segments internes et externes des photorécepteurs (Figure 2). Les potentiels évoqués visuels sont normaux de chaque coté. L'ERG plein champ montre des réponses scotopiques, mixtes et photopiques normales. L'ERG multifocal révèle une perte d'amplitude des réponses dans tous les secteurs aux deux yeux avec des réponses globales en dessous des normes.

## Discussion

La DMO est une pathologie dont le diagnostic est difficile. En effet, l’étiologie de la pathologie est encore méconnue bien qu'il semble qu'il y ait une transmission héréditaire de la maladie. L'atteinte rétinienne de la DMO est encore à ce jour mal précisée [1]. Actuellement, la clé du diagnostic de la DMO repose sur la mise en évidence d'anomalies à l'ERG multifocal alors que l'ERG plein champ, la fluoroangiographie et l'examen du FO sont tout à fait normaux. La dysfonction maculaire détectée par l'ERG multifocal et les résultats normaux de PEV, suggèrent une atteinte rétinienne distale aux cellules ganglionnaires [2]. Les clichés autofluorescents du fond d'oeil peuvent être utiles dans le diagnostic différentiel de la DMO avec certaines maculopathies telles que la maladie de Stargardt. En effet, ces maculopathies à leur stade précoce ne différent pas de la DMO sauf en ce qui concerne les images en autofluorescence qui révèlent des altérations de l'EPR que l'on ne retrouve pas dans la DMO. Dernièrement, certains auteurs ont montré chez des patients atteints de DMO, une diminution de l’épaisseur fovéolaire au T-D OCT associé à un amincissement de la couche nucléaire externe [3, 4]. Chez notre patiente, nous n'avons pas pu mettre en évidence cette observation avec le T-D OCT. Cependant, cet amincissement est retrouvé au S-D OCT et la nouvelle technique d'acquisition par cohérence optique de type “spectral-domain” offre une meilleure définition des structures rétiniennes, ce qui nous a permis de détecter un défect rétrofovéolaire au niveau de la jonction des segments interne et externes des photorécepteurs.

## Conclusion

Le diagnostic de la dégénérescence maculaire occulte est difficile. Il pourrait être facilité grâce aux nouvelles techniques d'acquisition des images par S-D OCT. De plus, ces nouvelles techniques apportent des informations utiles dans notre compréhension de cette pathologie rétinienne. Pour finir, il semble que les changements morphologiques visibles au S-D OCT soient corrélés à l'AV et à la progression de la maladie ce qui permettrait d’établir un pronostic quand au devenir dans le temps de la vision des patients atteints de DMO.
